# Acute kidney injury due to thrombotic microangiopathy in a patient with primary Sjögren’s syndrome

**DOI:** 10.1080/0886022X.2022.2087528

**Published:** 2022-06-17

**Authors:** Yi Wang, Xun Zhou, Xiaoyan Ma, Xinyu Yang, Yishu Wang, Min Tao, Binbin Cui, Tianyu Xiao, Shougang Zhuang, Na Liu

**Affiliations:** a Department of Nephrology, Shanghai East Hospital, Tongji University School of Medicine, Shanghai, China; b Department of Pathology, Shanghai East Hospital, Tongji University School of Medicine, Shanghai, China; c Department of Medicine, Rhode Island Hospital and Alpert Medical School, Brown University, Providence, RI, USA

##  

Dear Editor,

We described a case of monoclonal gammopathy–associated renal thrombotic microangiopathy (TMA) in a primary Sjögren’s syndrome (pSS) patient and who was successfully treated with a bortezomib-based regimen. A 66-years-old Chinese woman was admitted to our hospital with fatigue for a week and anuria for two days. Physical examination presented anemia and mild bilateral lower extremity edema. Old skin lesion could be seen in both lower limbs (Figure 1). The details of patient’s laboratory test results were listed in Table 1. Her hemoglobin (Hb) decreased from 79 g/L to 67 g/L and platelet (PLT) counts from 114 × 10^9^/L to 83 × 10^9^/L within five days accompanied by elevated lactate dehydrogenase (LDH) level of 1838 U/L. Her peripheral blood smear showed a small number of acanthocytes. Her blood urea nitrogen was 18.31 mmol/L, serum creatinine (Scr) was 653 μmol/L. Hypercoagulability workup revealed a high level of fibrinogen but negative anti-cardiolipin antibody immunoglobulin (Ig) G and IgM. Serum haptoglobin levels decreased from 146 to 7 mg/dL. Serum immunofixation electrophoresis detected monoclonal spikes of IgG and λ light chain. The free serum λ and κ light chain were both mildly elevated. The κ/λ-ratio was normal. Both complement C3 and C4 levels were depressed. Antinuclear antibodies, anti-neutrophil cytoplasmic antibodies, hepatitis virus, and anti-HIV-1/2 were all negative.

**Figure 1.**
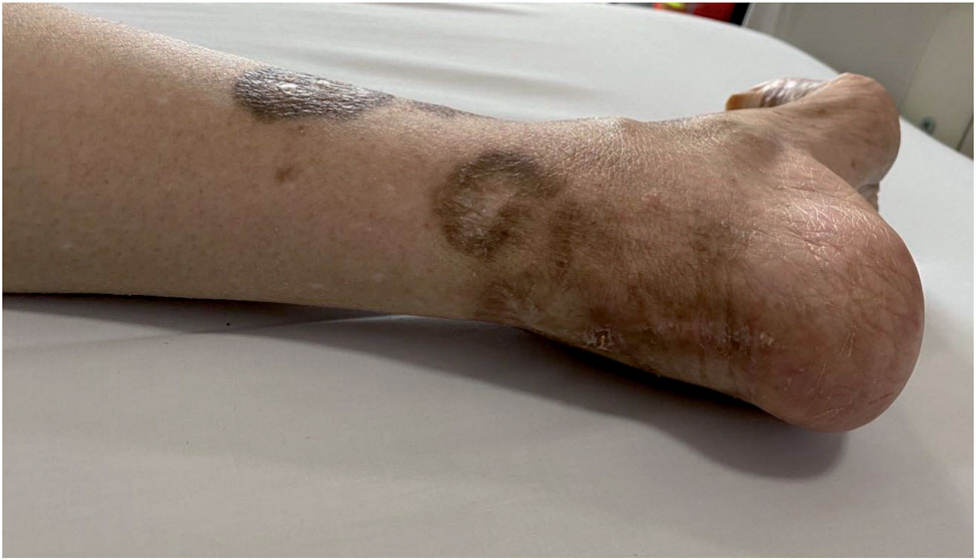
Skin injury. Skin necrosis scars on the foot and heel.

**Table 1.** The patient’s laboratory test results on admission.

Variables	Value	Normal range
Hemoglobin (g/L)	79	115–150
Platelet count (×10^9^/L)	114	125-350
Reticulocytes (%)	6.11	0.5-1.5
Serum albumin (g/L)	23	40-55
Blood urea nitrogen (mmol/L)	18.31	3.1-8.8
Serum creatinine (μmol/L)	653	41-81
Estimated glomerular filtration rate (ml/min /1.73 m^2^)	5	90-120
Lactate dehydrogenase (U/L)	1838	120-250
Fibrinogen (g/L)	6.07	2.38-4.98
D-dimer (mg/L)	7.07	0-0.5
Serum C3 (g/L)	0.569	0.7–1.4
Serum C4 (g/L)	<0.067	0.1-0.4
Haptoglobin (mg/dL)	146	32-205
Serum free κ chain (mg/L)	38.3	3.3–19.1
Serum free λ chain (mg/L)	51.6	5.71–26.3
Serum-free κ/λ chain ratio	0.74	0.26-1.65
Anti-cardiolipin Ab IgM (RU/mL)	2.44	<20
Anti-cardiolipin Ab IgG (RU/mL)	<2	<20
ANA (1:100)	neg	neg
ANA (1:320)	neg	neg
ANA (1:1000)	neg	neg
Anti-dsDNA (IU/mL)	1.25	0-10
Anti-Sm/RNP (RU/mL)	<2	<20
Anti-SSA Ab (RU/mL)	<2	<20
Anti-SSB Ab (RU/mL)	<2	<20

Abbreviations: ANA: antinuclear antibody; dsDNA: double-stranded DNA; anti-Sm/RNP: anti-Smith/ribonucleoprotein; anti-SSA Ab: anti-Sjögren’s syndrome A antibody; anti-SSB Ab: anti-Sjögren’s syndrome B antibody.

Four years ago, she was diagnosed with pSS based on myalgia, dry mouth and positive labial salivary gland biopsy. Since then, she took oral 10 mg of prednisone daily. Two months later, she was administered intravenous 0.8 g of cyclophosphamide (CTX) every two months because of elevated erythrocyte sedimentation rate and lower extremity skin necrosis. Currently, the total cumulative dose of CTX was 18.2 g. Two years ago, the patient’s serum immunofixation electrophoresis revealed a monoclonal protein with an IgG-λ. However, bone marrow (BM) biopsy did not find any malignant hematological diseases at that time. Two months ago, she developed skin necrosis on bilateral lower extremity again and was administered with oral prednisone 50 mg daily without adjustment of the CTX treatment plan. The skin lesions gradually improved and the dose of prednisone was reduced to 20 mg daily finally. Her renal function was quite normal until she went to the community hospital for routine examination last week.

Renal biopsy showed many red blood cells gathered in the glomeruli and arterioles, obstructing the lumen with massive thrombosis, indicating renal TMA (Figure 2). No immune deposits were shown by immunofluorescence microscopy or electron microscopy. We performed hemodialysis and initiated therapeutic plasma exchange (PE) (on hospital days 7, 14) in conjunction with low-molecular-weight heparin (4000 U once every other day). Later on, we ceased PE because of the activity of the von Willebrand factor-cleaving protease ADAMTS13 was 61.37% (normal 42.16–126.37%) and anti-ADAMTS13 IgG was negative. The BM biopsy showed plasma cells was 8% and flow cytometry indicated monoclonal plasma cells accounts for 1.2%. The patient then received Bortezomib 0.8 mg/m^2^ (d1, d8) subcutaneous injection every month. During this period, we changed hemodialysis into continuous ambulatory peritoneal dialysis (CAPD) and gradually reduce the dose of prednisone to 20 mg daily. The patient recovered from anuria three weeks after admission. When she was discharged, her urine volume had increased to 1000 mL/24h and the Hb was 92 g/L, PLT was 118 × 10^9^/L, LDH was 467 U/L, and Scr was 326 μmol/L. The overall course of disease evolution was shown in Figure 3.

**Figure 2.**
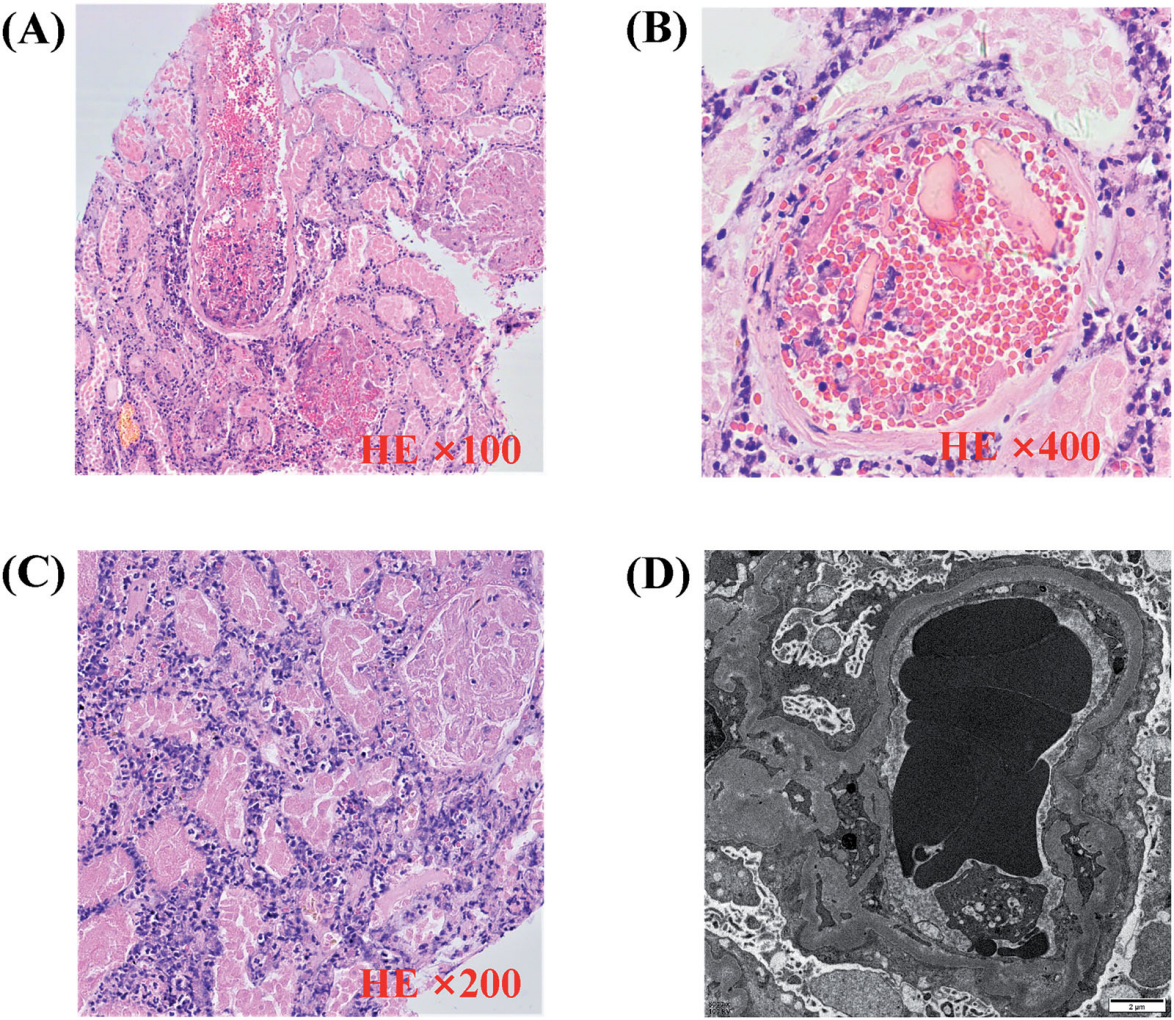
Pathology of renal biopsy. (A) Light microscopy (LM) shows small artery containing abundant erythrocytes (HE ×100). (B) LM shows capillary loops containing abundant erythrocytes. Renal interstitium is infiltrated with many neutrophils and mononuclear cells (HE ×400). (C) Tubular epithelial cell disintegrant and brush border loss, tubular basement membranes are exposed. Tubular cavity containing abundant coagulation necrotic material (HE ×200). (D) Electron microscopy demonstrates diffuse subendothelial widening by flocculent material. There is also diffuse fusion of podocyte foot processes, but no electron dense deposits are seen; erythrocytes accumulate in glomerular capillary (×12 000).

**Figure 3.**
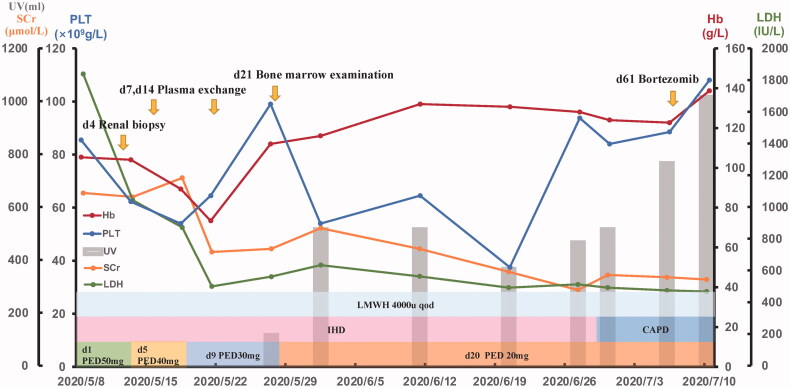
The patient’s treatment follow-up graph. The graph displays the main clinic indexes including hemoglobin, platelets, lactate dehydrogenase, creatinine, and urine volume. The treatment interventions during the hospital course are showed above. CAPD: continuous ambulatory peritoneal dialysis; Hb: hemoglobin; IHD: intermittent hemodialysis; LDH: lactate dehydrogenase; LMWH: low-molecular-weight heparin; PED: prednisone; PLT: platelets; Scr: serum creatinine; UV: urine volume.

## Discussion

TMA is characterized by a group of clinical manifestations including thrombocytopenia, microvascular hemolytic anemia (MAHA) and organ damage mediated by endothelial injury. TMA syndrome can be classified into: thrombotic thrombocytopenic purpura (TTP) mediated by acquired or inherited ADAMTS13 deficiency; the hemolytic–uremic syndrome (HUS) triggered by Shiga toxin; atypical HUS (aHUS) mediated by acquired or hereditary complement dysregulation; and drug-induced TMA [1]. Renal TMA is most often observed in patients with complement-mediated and drug-induced TMA, and usually presents as acute kidney injury (AKI), hypertension, mild thrombocytopenia, and MAHA [1].

In this case, the main clinical finding was AKI, mild thrombocytopenia, and MAHA which defined by schistocytosis, elevated LDH levels, and decreased haptoglobin. Since there were no intestinal infections or ADAMTS13 deficiency, the possible diagnosis was of an aHUS or drug-induced TMA. Hereditary aHUS was excluded since family history was negative. Then the possible triggers for acquired aHUS included monoclonal IgG-λ, history of pSS and medication. There is a high prevalence of monoclonal gammopathy in patients with TMA. A retrospective study showed the prevalence of monoclonal gammopathy was 13.7% in adults with a clinical diagnosis of TMA, even up to 21% among patients aged 50 years and more [2]. Monoclonal gammopathy injured renal by depositing in the kidney tissue directly or by an indirect mechanism *via* dysregulation of the alternative pathway of complement, so TMA was now attributed to one of the kidney injuries associated with monoclonal gammopathy of renal significance (MGRS) [3]. Autoantibody activity of the monoclonal immunoglobulins against ADAMTS13 or complement factor H has been demonstrated [4]. According to her extremely low complement C4 levels and negative renal immunofluorescence, we propose that the monoclonal gammopathy caused complement lectin pathway dysregulation which subsequently induced complement-mediated aHUS.

Sjögren’s syndrome has been well recognized as a crossroad between autoimmunity and lymphoproliferation. Patients with pSS have a 14-fold enhanced risk of developing lymphomas compared to general population [5]. As the resulting from a clonal proliferation of plasma cells or B lymphocytes, pSS is one of the best examples of non-hematological diseases which often present with monoclonal gammopathy. It was reported that monoclonal Ig (MIg) was detected in 22% of patients with pSS, and MIg G was the most frequent type of band detected [6]. However, there was no evidence of myeloma or other hematological malignancies despite mildly elevated proportion of plasma cells in her BM biopsy. It was necessary to pay attention to the possibility of the patient’s future progression into myeloma.

Drug is another common etiology of TMA. Al-Nouri et al. systematically searched for all published reports of drug-induced TMA and identified 22 drugs with a definite causal association with TMA [7]. Quinine, cyclosporine and tacrolimus were the three drugs with the most evidence. Drug-induced TMA may be characterized by a sudden onset of severe systemic symptoms mediated by drug-dependent antibody or a dose-related gradual onset of renal failure occurs over weeks or months [1]. In our case, the patient had neither progressive renal failure nor newly administered drugs before the onset of AKI, so we excluded the diagnosis of drug-induced TMA.

The standard treatment of TMA associated with monoclonal gammopathy remains unknown. With the goal of eradicating the clonal cells producing the immunoglobulin, bortezomib-based regimens for a non-IgM monoclonal immunoglobulin was recommended [8]. Clone-targeted chemotherapy might improve clinical parameters in monoclonal gammopathy-associated TMA was currently reported. Cheungpasitporn et al. reported a case of successful treatment with bortezomib, lenalidomide, and dexamethasone (VRD) regimen for a patient with aHUS and monoclonal protein refractory to eculizumab therapy [9]. The patient had a significant improvement in proteinuria, renal function and MAHA since starting VRD. The efficacy of bortezomib in the present case confirmed the possible mechanism that monoclonal gammopathy may be associated with TMA *via* complement dysregulation.

## Conclusion

TMA may be mediated by monoclonal gammopathy *via* complement dysregulation. bortezomib-based regimens could be considered as available treatment options.

## References

[1] George JN, Nester CM. Syndromes of thrombotic microangiopathy. N Engl J Med. 2014;371(7):654–666. 25119611 10.1056/NEJMra1312353

[2] Ravindran A, Go RS, Fervenza FC, et al. Thrombotic microangiopathy associated with monoclonal gammopathy. Kidney Int. 2017;91(3):691–698. 27998645 10.1016/j.kint.2016.09.045

[3] Sethi S, Rajkumar SV, D'Agati VD. The complexity and heterogeneity of monoclonal immunoglobulin-associated renal diseases. J Am Soc Nephrol. 2018;29(7):1810–1823. 29703839 10.1681/ASN.2017121319 PMC6050917

[4] Bridoux F, Cockwell P, Glezerman I, et al. Kidney injury and disease in patients with haematological malignancies. Nat Rev Nephrol. 2021;17(6):386–401. 33785910 10.1038/s41581-021-00405-7

[5] Liang Y, Yang Z, Qin B, et al. Primary Sjogren’s syndrome and malignancy risk: a systematic review and meta-analysis. Ann Rheum Dis. 2014;73(6):1151–1156. 23687261 10.1136/annrheumdis-2013-203305

[6] Brito-Zerón P, Retamozo S, Gandía M, et al. Monoclonal gammopathy related to Sjögren syndrome: a key marker of disease prognosis and outcomes. J Autoimmunity. 2012;39(1-2):43–48. 22297146 10.1016/j.jaut.2012.01.010

[7] Al-Nouri ZL, Reese JA, Terrell DR, et al. Drug-induced thrombotic microangiopathy: a systematic review of published reports. Blood. 2015;125(4):616–618. 25414441 10.1182/blood-2014-11-611335 PMC4304106

[8] Sethi S, Rajkumar SV. Monoclonal gammopathy-associated proliferative glomerulonephritis. Mayo Clin Proc. 2013;88(11):1284–1293. 24182705 10.1016/j.mayocp.2013.08.002

[9] Cheungpasitporn W, Leung N, Sethi S, et al. Refractory atypical hemolytic uremic syndrome with monoclonal gammopathy responsive to bortezomib-based therapy. Clin Nephrol. 2015;83(6):363–369. 25345382 10.5414/CN108363

